# Altered intestinal microbiota in children with bronchiolitis

**DOI:** 10.3389/fmicb.2023.1197092

**Published:** 2023-06-15

**Authors:** Xiao-bin Wu, Jian Wang, Yuan Tang, Jing Jiang, Xue-mei Li

**Affiliations:** ^1^Chongqing Health Center for Women and Children, Chongqing, China; ^2^Women and Children's Hospital of Chongqing Medical University, Chongqing, China

**Keywords:** bronchiolitis, intestinal microbiota, metabolism, Clostridium, Sphingomonas

## Abstract

**Objective:**

To investigate the correlation between the alteration of intestinal microbiota and disease in children with bronchiolitis.

**Methods:**

Fifty seven children diagnosed with bronchiolitis from January 2020 to January 2022 in our pediatric department were included as the case group, and another 36 normal children were included as the control group. Stool and blood were collected from both groups for high-throughput sequencing, untargeted metabolite detection and ELISA. A mouse model of RSV infection was established to validate the results of clinical case detection.

**Results:**

Body weight, passive smoking, and a host of other factors were possible as acute bronchiolitis influencing factors in the onset of acute bronchiolitis. The alpha diversity Shannon, Simpson and Pielou’s evenness indices were significantly lower in children with acute bronchiolitis than in healthy children with gated levels of Firmicutes, Bacteroidetes and genus levels of Clostridium and other short chain fatty acid-producing bacteria. The relative abundance of short-chain fatty acid (SCFAs)-producing bacteria decreased and the abundance of genus-level sphingolipid-producing bacteria Sphingomonas increased; the progression of acute bronchiolitis is likely to be associated with the abundance of Clostridium and Sphingomonas and higher fecal amino acid concentrations, including FF-MAS, L-aspartic acid, thioinosinic acid, picolinic acid; supplementation with *Clostridium butyricum* significantly alleviated RSV infection-induced lung inflammation.

**Conclusion:**

The progression of bronchiolitis may be associated with altered intestinal microbiota, decreased SCFAs and elevated sphingolipids metabolism in children. Some fecal bacteria and metabolites may predict the onset of bronchiolitis, and oral administration of *Clostridium butyricum* may alleviate RSV infection-induced pulmonary inflammation.

## 1. Introduction

Bronchiolitis is the most common lower respiratory tract infection in infancy and childhood, with wheezing as the main clinical manifestation, with an average incidence of approximately 20% (Caffrey Osvald and Clarke, 2016). The 2018 Australasian Guidelines for the management of bronchiolitis state that 56% of all hospital admissions in Australia and one in three children of the same age in New Zealand have a higher rate of hospitalization of children with bronchiolitis in the Asia-Pacific region, including China, than in developed countries (Cavaye et al., 2019). Bronchiolitis not only has a high incidence and hospitalization rate but also has a high mortality rate and is closely associated with recurrent wheezing and childhood asthma later in life (Garcia-Garcia et al., 2016). Respiratory viral infections, particularly respiratory syncytial virus (RSV) and rhinovirus, are the most importance risk factors for the onset of bronchiolitis in infants and small children. Although the exact mechanism of RSV induced pathogenesis is not clear, the relative superiority of Th2 cytokines over Th1 cytokines has been shown to be critical for the occurrence of asthma and exacerbation of asthma caused by RSV infection (Bueno et al., 2008; Russell et al., 2017). As the incidence of bronchiolitis is increasing year by year, the health of children is seriously affected (Azzari et al., 2021). Therefore, it is of great scientific and clinical importance to investigate the pathogenesis of bronchiolitis and to find new treatment methods the disease.

In recent years, with the development of bioinformatics and biotechnology, people have begun to pay attention to the relationship between intestinal microbiota and lung diseases and have proposed the concept of the “microbiota-gut-lung axis” (Budden et al., 2017). The intestine is the largest immune organ of the human body and houses a very rich number and variety of intestinal microbiota. There are more than 1,000 kinds of intestinal microbiota known, accounting for 80% of the total microorganisms in the human body (Sender et al., 2016). There is increasing evidence that intestinal microorganisms not only influence the internal environment and function of the intestine but also participate in the occurrence and development of various respiratory diseases through barrier function, intestinal mucosal immunity, and regulation of intestinal microbial metabolites (Leimena et al., 2013). The intestinal microbiota and the respiratory tract Regarding the correlation between intestinal microbiota and respiratory diseases, especially airway allergic diseases, the main focus is on the association of intestinal microbiota with asthma and allergic rhinitis (Zhang et al., 2018). However, there are few reports on acute bronchiolitis, a wheezing disease in infants and children, before the onset of asthma. In this study, we intend to investigate the relationship between changes in intestinal microbiota and metabolites and disease in children with bronchiolitis by performing high-throughput sequencing and untargeted metabolite analysis in the stools of 57 children with bronchiolitis collected in our hospital and 36 healthy children during the same period.

## 2. Materials and methods

### 2.1. Clinical information

(1) 57 children diagnosed with bronchiolitis from January 2020 to January 2022 in the pediatric department of Chongqing Maternal and Child Health Hospital were included in this study as the case group, and 36 age-and sex-matched normal children were included as the control group. The study was discussed and approved by the ethics committee of Chongqing Maternal and Child Health Hospital, and all parents of the children signed the informed consent form.
(2) Inclusion criteria for children with bronchiolitis: the diagnostic criteria of the Expert Consensus on Diagnosis, Treatment and Prevention of bronchiolitis (2014 version) of the Respiratory Group of the Pediatric Branch of the Chinese Medical Association were followed. All children were diagnosed according to their medical history, physical examination and viral tests, including 44 cases (77.2%) of RSV virus infection and 13 cases (22.2%) of rhinovirus infection, aged from 3 months to 2 years, with normal blood counts and C-reactive protein tests.
(3) Exclusion criteria for children with bronchiolitis: those with obvious toxic symptoms and extrapulmonary complications, those with severe malnutrition, those with serious diseases of other systems, those with mental abnormalities that do not cooperate with treatment or affect the evaluation of efficacy, those with immunodeficiency, those with diarrhea or probiotic preparations within the last 2 months, those who used antibiotics 1 week before enrollment, those who received glucocorticoids, antihistamines, and immunomodulators 2 weeks before enrollment, those born at <32 weeks of age, and those who were allergic to therapeutic drugs.
(4) Inclusion criteria for healthy children: normal children aged 3 months to 2 years who underwent a health checkup at the hospital during the same period.
(5) Exclusion criteria for healthy children: those who had diarrhea or probiotic preparations within the last 2 months, those who had any respiratory, digestive and allergic diseases in the last 1 month, those who used antibiotics 1 week before enrollment, those who received glucocorticoids, antihistamines, or immunomodulators 2 weeks before enrollment, those who had a previous history of bronchiolitis or asthma, and those whose gestational age at birth was <32 weeks.
(6) Clinical data collection: All enrolled children were evaluated by clinicians, and basic clinical data were collected after successful enrollment, including residence status, birth status, previous illnesses, history of allergic diseases, breastfeeding history, parental smoking history, household income, and parental history of allergic diseases.
(7) BMI measurement methods: a lever scale with a maximum load of 50 kg was used. The scale was calibrated before measurement, and the child was weighed after they had defecated and was dressed in light clothing; weight was recorded to 2 decimal places in kilograms. Height measurement: a vertical height meter was used, and the child was measured twice in the morning and evening. BMI was calculated as BMI = weight (kg)/height weight (kg)/height (m2), and the mean value was taken.
(8) Child weight-for-height z-scores and weight-for-length categories: the statistician calculated infant weight for length z-scores using a SAS® macro from the U.S. Centers for Disease Control and Prevention (CDC) that draws on reference values from the World Health Organization growth data for healthy infants (Hendrickson and Pitt, 2022). The analysis used Z-cores measured at previous hospital visits to classify infants into four weight-for-length categories: underweight (*z* < −1.28, < 10th percentile); normal weight (−1.28 ≤ *z* < 1.04, 10th to <85th percentile); overweight (1.04 ≤ *z* < 1.64, 85th to <95th percentile); and obese (*z* ≥ 1.64, ≥ 95th percentile). From these categories, the statistician created a dichotomous (normal or underweight = 0; overweight or obese = 1) indicator of weight-for length status.

### 2.2. Sample collection and processing

(1) Intestinal microbiota DNA extraction and amplification: All stool samples were collected on site by nurses, collected and stored frozen at −80°C. Bacterial genomic DNA samples were extracted using a Fast DNA SPIN extraction kit (MP Biomedicals, Santa Ana, CA, USA) according to the instructions for use. The quantity and quality of the extracted DNA were measured using a NanoDrop ND-1000 spectrophotometer (Thermo Fisher Scientific, Waltham, MA, USA) and agarose gel electrophoresis, respectively. Ribosomal RNA contains multiple conserved and highly variable regions, and we typically use conserved regions to design primers to amplify single or multiple variable regions of rRNA genes, which are then sequenced to analyze microbial diversity. In this experiment, the highly variable V3 and V4 region of the bacterial 16S rRNA gene with a length of approximately 468 bp was used for sequencing. PCR was performed using NEB Q5 DNA high-fidelity polymerase, and the required components for the PCR were configured in the PCR column. After the components of the PCR were configured, the template DNA was denatured for 30 s at 98°C on a PCR instrument and then entered into the amplification cycle. In each cycle, the template was denatured by holding at 98°C for 15 s, and then the temperature was lowered to 50°C and held for 30 s to allow primers to fully anneal to the template; the temperature was held for 30 s at 72°C to allow primers to extend over the template and synthesize DNA, completing a cycle. This cycle was repeated 25–27 times to allow a large accumulation of amplified DNA fragments. Finally, the product was kept at 72°C for 5 min to allow for complete extension and stored at 4°C. The amplification results were subjected to 2% agarose gel electrophoresis, and the target fragments were excised and then recovered using an Axygen Gel Recovery Kit.
(2) Amplification product purification and recovery: (1) First, 25 μL of PCR product was added to 0.8 times the volume of magnetic beads (Vazyme VAHTSTM DNA Clean Beads), and the tube was shaken to fully suspend the beads. The tube was then placed on a magnetic rack for 5 min, and the supernatant was carefully aspirated with a pipette gun. (2) Then, 20 μL of 0.8 times the bead washing solution was added, and the tube was shaken to fully suspend the beads. The tube was then placed on a magnetic rack for 5 min, and the supernatant was carefully aspirated out; (3) Then, 200 μL 80% ethanol was added, and the tube was placed in reverse on the magnetic rack so that the beads would be adsorbed to the other side of the PCR tube, and the supernatant was then aspirated out. (4) The tube was placed at room temperature and allowed to rest for 5 s to allow complete alcohol volatilization, with cracks possibly appearing in the magnetic beads; (5) Then, 25 μL Elution Buffer was added to elute the products. (6) The PCR tube was placed on the adsorption rack for 5 min to allow the beads to fully adsorb, and the supernatant was removed to a clean 1.5 mL centrifuge tube for storage. The resulting product was sent to Shanghai Paaser Biotech for high-throughput sequencing.
(3) Metabolite extraction: After the sample was ground in liquid nitrogen, 400 μL of precooled methanol/acetonitrile/water solution (4:4:2, v/v) was added to the sample, vortexed and mixed, left at −20°C for 60 min, and centrifuged at 14,000 × g for 20 min at 4°C. The supernatant was dried under vacuum, and 100 μL of aqueous acetonitrile solution (acetonitrile:water = 1:1, v/v) was added to the sample for mass spectrometry, vortexed, and centrifuged at 14,000 × g for 15 min at 4°C. The supernatant was removed and sent to Shanghai Paisano Biotech for LC–MS/MS analysis.

### 2.3. Sequence processing

Paired-end (paired-end) sequencing of community DNA fragments was performed using the Illumina platform. (1) First, the raw downstream data of high-throughput sequencing were initially screened according to sequence quality; any problematic samples were retested and complemented. (2) The raw sequences that passed the initial quality screening were divided into libraries and samples according to index and barcode information, and barcode sequences were removed. (3) Sequence denoising or OTU clustering was performed according to the QIIME2 dada2 analysis process or Vsearch software’s analysis process. (4) The specific composition of each sample (group) at different species taxonomic levels was demonstrated to understand the overall profile. (5) The alpha diversity level of each sample was assessed based on the distribution of ASVs/OTUs in different samples, and the appropriateness of the sequencing depth was reflected by sparse curves. (6) At the ASV/OTU level, the distance matrix of each sample was calculated, and the difference in beta diversity and the significance of the difference between different samples (groups) were measured by various unsupervised rankings and clustering means, combined with the corresponding statistical tests. (7) At the level of taxonomic composition of species, we further measured the differences in species abundance composition among samples (groups) by various unsupervised and supervised ranking, clustering and modeling means, combined with corresponding statistical tests, and tried to find the marker species. (8) Association networks were constructed, topological indices were calculated, and the identification of keystone species was attempted based on the compositional distribution of species in each sample.

### 2.4. Metabolite extraction

After the sample was ground in liquid nitrogen, 400 μL of precooled methanol/acetonitrile/water solution (4:4:2, v/v) was added to the sample, vortexed and mixed, left at −20°C for 60 min, and centrifuged at 14,000 × g for 20 min at 4°C. The supernatant was dried under vacuum, and 100 μL of aqueous acetonitrile solution (acetonitrile:water = 1:1, v/v) was added to the sample for mass spectrometry, vortexed, and centrifuged at 14,000 × g for 15 min at 4°C. The supernatant was then dried under vacuum.

### 2.5. LC–MS/MS analysis and metabolite data analysis

(1) Chromatographic conditions: The separation was performed on an ACQUITY UPLC BEH C18 column (100 mm*2.1 mm, 1.7 μm, Waters, USA) with a column temperature of 40°C and a flow rate of 0.3 mL/min, in which the A mobile phase was water and 0.1% formic acid and the B mobile phase was acetonitrile. The metabolites were eluted using the following gradients: 0–0.5 min, 5% B; 0.5–1.0 min. The loading volume of each sample was 5 μL. The samples were placed in an autosampler at 4°C throughout the analysis. To avoid the effects caused by fluctuations in the instrument detection signal, a random order was used for continuous analysis of the samples. QC samples were inserted after each group of samples in the sample queue for monitoring and evaluating the stability of the system and the reliability of the experimental data.
(2) Mass spectrometry conditions: electrospray ionization (ESI) positive and negative ion modes were used for detection. The samples were separated by UHPLC and analyzed by mass spectrometry using a Q-Exactive quadrupole-electrostatic field orbital trap high-resolution mass spectrometer (Thermo Fisher Scientific).

#### 2.5.1. Metabolite data analysis

(1) The raw data obtained from mass spectrometry acquisition were processed by Compound Discoverer 3.0 (Thermo Fisher Scientific) software for peak extraction, peak alignment, peak correction, normalization and other data preprocessing. Metabolite structure identification was performed by exact mass number matching (<25 ppm) and secondary spectrum matching, searching the laboratory’s own database as well as Bio cyc, HMDB, metlin, HFMDB, Lipidmaps and other databases. SIMCA-P 14.1 software (Umetrics, Umea, Sweden) was applied for pattern recognition, and the data were preprocessed by Pareto scaling and subjected to multidimensional statistical analysis, including unsupervised principal component analysis (PCA), supervised partial least squares discriminant analysis (PLS-DA) and orthogonal partial least squares discriminant analysis (OPLS-DA). Unidimensional statistical analyses included Student’s t test and multiplicative analysis of variance. R software was used to construct volcano plots. (2) The OPLS-DA model VIP > 1 and *p* value <0.05 were used as criteria to screen for significant differential metabolites, followed by cluster analysis and KEGG metabolic pathway analysis for differential metabolites.

### 2.6. Establishment of the RSV-infected mouse model

(1) Preparation of mice: BALB/c mice, SPF grade, male and female, 6–8 weeks old, provided by the Animal Experiment Center of Chongqing Medical University, were kept in an SPF room, and feed and drinking water were sterilized by high temperature and pressure. A total of 12 mice were randomly divided into 2 groups (experimental group and control group) with 6 mice in each group. To avoid the interaction of intestinal microorganisms, each mouse was housed in a single cage. (2) Hep-2 cell culture and RSV propagation: Hep-2 cells and the RSV A2 strain were purchased from ATCC. After the cell monolayer was spread on nearly 90% of the bottom of the culture flask, RSV was inoculated, and the old solution was discarded after decolonization at room temperature. DMEM containing 10% fetal bovine serum was added, the cells were transferred to a 37°C incubator with 5% CO2, and syncytial lesions were continuously observed. When cell fusion reached 90–100%, the viral fluid was collected and centrifuged at 4°C and 12,000 RPM for 10 min, and the supernatant was separated on ice and quickly transferred to −80°C for storage. The titer of RSV A2 used in this study was 1.5×10^8^ PFU/ml, as determined by the Plaque Assay. (3) Establishment of the RSV infection animal model (mouse bronchiolitis): At 6–8 weeks, female BALB/c mice were divided into RSV-infected and control groups. On Day 5, the mice were sacrificed, and lung specimens were collected. (4) Mice in the probiotic treatment group: *Clostridium butyricum* (BNCC, 337,239; 200 μL/day, concentration of live bacteria, 1 × 10^8^ CFU/mL) was administered via the intragastric route for 5 consecutive days in conjunction with RSV infection (Zhu et al., 2021). Meanwhile, the control group was administered via the intragastric route using placebo maltodextrin.

### 2.7. Lung histopathology section processing

(1) Specimen fixation, dehydration, and embedding: The fresh left lung tissue of mice was fixed with 10% neutral formaldehyde solution for more than 24 h, after which it was removed and placed in an embedding box, rinsed with tap water overnight, and dehydrated in an automatic dehydration machine for 8–10 h. The specimen was removed from the embedding box and placed in an embedding tank, and paraffin was embedded to form a tissue wax block. (2) Slicing and staining: The tissue wax block was cut into 4-to 5-μm-thick sections with a slicing machine, placed on a poly-l-lysine-coated slide, placed in an oven at 60°C to fully melt the paraffin wax, and subjected to HE staining. The specific operation steps were as follows: xylene I dewaxing for 5 min, xylene II dewaxing for 10 min, 100% ethanol for 3 min, 95% ethanol for 2 min, 75% ethanol for 3 min, 5 min tap water rinse, 8–10 min hematoxylin staining, 3–5 s hydrochloric acid alcohol soak, 2 min tap water rinse, 3 s saturated lithium carbonate soak, 5 min tap water rinse, 95% ethanol 1 min, 1–2 min eosin staining, 5 min tap water wash, 95% ethanol 30 s, 100% ethanol 10 s, and 100% ethanol 10 s, after which the samples were dried, and neutral resin was applied to seal the film. (3) Histomorphometric analysis: The inflammation scoring was performed according to the Myou et al. (2003) method to assess the histopathological inflammatory changes in the lung.

### 2.8. Serum inflammatory factor assay

Venous blood was drawn from the children in dry tubes and centrifuged at 3,000 r/min for 15 min to separate the serum, and the supernatant in the frozen tubes was stored at −80°C. IL-4 (abcam, ab84269, and ab9622), IL-5 (abcam, ab242707, and ab9624), TNF-α (abcam, ab242949 and ab9979) and IgE (abcam, ab302738 and ab195580) were detected by ELISA according to the instructions provided with the kit.

### 2.9. Statistical analysis

To correct for multiple comparisons, the two-stage linear step-up procedure of Benjamini, Krieger, and Yekutieli *post hoc* tests was used to control the false discovery rate (FDR). A *p* value <0.05 or FDR-corrected p (q) <0.05 was considered statistically significant. Microbial analyses, including microbial community composition and diversity analyses, were performed by R 3.6.1 (the R Foundation for Statistical Computing, Vienna, Austria), and the linear discriminant analysis effect size (LEfSe) analysis was implemented by the LEfSe tool.^1^ The functional prediction results of the intestinal microbiota were obtained based on the microbial functional gene profile in the Kyoto Encyclopedia of Genes and Genomes (KEGG).^2^ Serum metabolomic data were analyzed after Pareto scaling and log transformation. Principal component analysis (PCA), latent structure orthogonal-discriminant analysis (OPLS-DA), and pathway enrichment analysis were implemented on the MetaboAnalyst 5.0 platform, a web-based metabolomics pathway analysis platform.^3^ The visualization was performed with R 3.6.1. The pathway map was analyzed with CytoScape and MetaMapp (Boston, MA, USA). Finally, we performed a multifactorial ANOVA to examine the effect of the variables in Table 1 on the relative abundance results at the genus level for the two groups (Gloor et al., 2017; Table 2).

**Table 1:** Analysis of clinical data of the two groups.

Factors	Healthy children	Bronchiolitis	*p* value
Sex			0.833
Male	20	33	
Female	16	24	
Age			0.6576
<1 year old	22	36	
≥1 year old	14	21	
Age (<1 year old)			0.1726
3–6 months old	8	19	
6–12 months old	14	17	
Body weight			0.0005
Normal	26	20	
Obesity	10	37	
Pets			0.0616
Yes	30	38	
No	6	19	
Passive Smoking			0.0255
Yes	14	30	
No	22	27	
Family history of allergies			<0.0001
Yes	5	40	
No	31	17	
Siblings			0.1558
Yes	29	37	
No	7	20	
Place of residence			0.0081
City	25	52	
Rural	11	5	
Eczema			0.0091
Yes	15	41	
No	21	16	
Antibiotics in the neonatal period			<0.0001
Yes	11	48	
No	25	9	
Household income			0.6274
<100,000	26	44	
>100,000	10	13	
Breastfeeding >3 months			0.0012
Yes	25	19	
No	11	38	
*In vitro* fertilization			0.0412
Yes	3	14	
No	33	43	

**Table 2:** A multifactorial ANOVA and the results showed that the variables in Table 1 did not have a significant effect on the genus level relative to the abundance of the 2 groups compared.

Multivariate tests^a^						
Effect		Value	*F*	Hypothesis df	Error df	Sig.
Intercept	Pillai’s Trace	0.998	496.967^b^	47.000	38.000	0.000
	Wilks’ Lambda	0.002	496.967^b^	47.000	38.000	0.000
	Hotelling’s Trace	614.670	496.967^b^	47.000	38.000	0.000
	Roy’s Largest Root	614.670	496.967^b^	47.000	38.000	0.000
Passive smoking	Pillai’s Trace	0.635	1.406^b^	47.000	38.000	0.140
	Wilks’ Lambda	0.365	1.406^b^	47.000	38.000	0.140
	Hotelling’s Trace	1.739	1.406^b^	47.000	38.000	0.140
	Roy’s Largest Root	1.739	1.406^b^	47.000	38.000	0.140
Family history of allergies	Pillai’s Trace	0.611	1.269^b^	47.000	38.000	0.226
	Wilks’ Lambda	0.389	1.269^b^	47.000	38.000	0.226
	Hotelling’s Trace	1.570	1.269^b^	47.000	38.000	0.226
	Roy’s Largest Root	1.570	1.269^b^	47.000	38.000	0.226
Place of residence	Pillai’s Trace	0.451	0.665^b^	47.000	38.000	0.908
	Wilks’ Lambda	0.549	0.665^b^	47.000	38.000	0.908
	Hotelling’s Trace	0.823	0.665^b^	47.000	38.000	0.908
	Roy’s Largest Root	0.823	0.665^b^	47.000	38.000	0.908
Eczema	Pillai’s Trace	0.655	1.533^b^	47.000	38.000	0.088
	Wilks’ Lambda	0.345	1.533^b^	47.000	38.000	0.088
	Hotelling’s Trace	1.896	1.533^b^	47.000	38.000	0.088
	Roy’s Largest Root	1.896	1.533^b^	47.000	38.000	0.088
Antibiotics in the neonatal period	Pillai’s Trace	0.422	0.589^b^	47.000	38.000	0.957
	Wilks’ Lambda	0.578	0.589^b^	47.000	38.000	0.957
	Hotelling’s Trace	0.729	0.589^b^	47.000	38.000	0.957
	Roy’s Largest Root	0.729	0.589^b^	47.000	38.000	0.957
Breastfeeding 3months	Pillai’s Trace	0.492	0.783^b^	47.000	38.000	0.788
	Wilks’ Lambda	0.508	0.783^b^	47.000	38.000	0.788
	Hotelling’s Trace	0.969	0.783^b^	47.000	38.000	0.788
	Roy’s Largest Root	0.969	0.783^b^	47.000	38.000	0.788
*In vitro* fertilization	Pillai’s Trace	0.525	0.895^b^	47.000	38.000	0.644
	Wilks’ Lambda	0.475	0.895^b^	47.000	38.000	0.644
	Hotelling’s Trace	1.107	0.895^b^	47.000	38.000	0.644
	Roy’s Largest Root	1.107	0.895^b^	47.000	38.000	0.644
Bronchiolitis	Pillai’s Trace	0.768	2.675^b^	47.000	38.000	0.001
	Wilks’ Lambda	0.232	2.675^b^	47.000	38.000	0.001
	Hotelling’s Trace	3.308	2.675^b^	47.000	38.000	0.001
	Roy’s Largest Root	3.308	2.675^b^	47.000	38.000	0.001

^a^Design: Intercept + PassiveSmoking + Familyhistoryofallergies + Placeofresidence + Eczema + Antibioticsintheneonatalperiod + Breastfeeding3months + Invitrofertilization + bronchiolitis.

^b^Exact statistic.

## 3. Results

### 3.1. Analysis of clinical data

There were no significant differences in sex characteristics and age between the two groups of children included in the study, and there were no significant differences between the two groups regarding the presence of pets in the family (*p* = 0.0616), family economic income (*p* = 0.6274), and the presence of siblings in the family (*p* = 0.1558). However, there were significant differences between the two groups in weight (*p* = 0.0005), passive smoking (*p* = 0.0255), allergic family history (*p* < 0.0001), family residence (*p* = 0.0081), presence of eczema (*p* = 0.0091), breastfeeding >3 months (*p* = 0.0012), and whether conception occurred *via in vitro* fertilization (*p* = 0.0412), as detailed in Table 1.

### 3.2. Alpha and beta diversity analyses of the two sample groups

**Figure 1:**
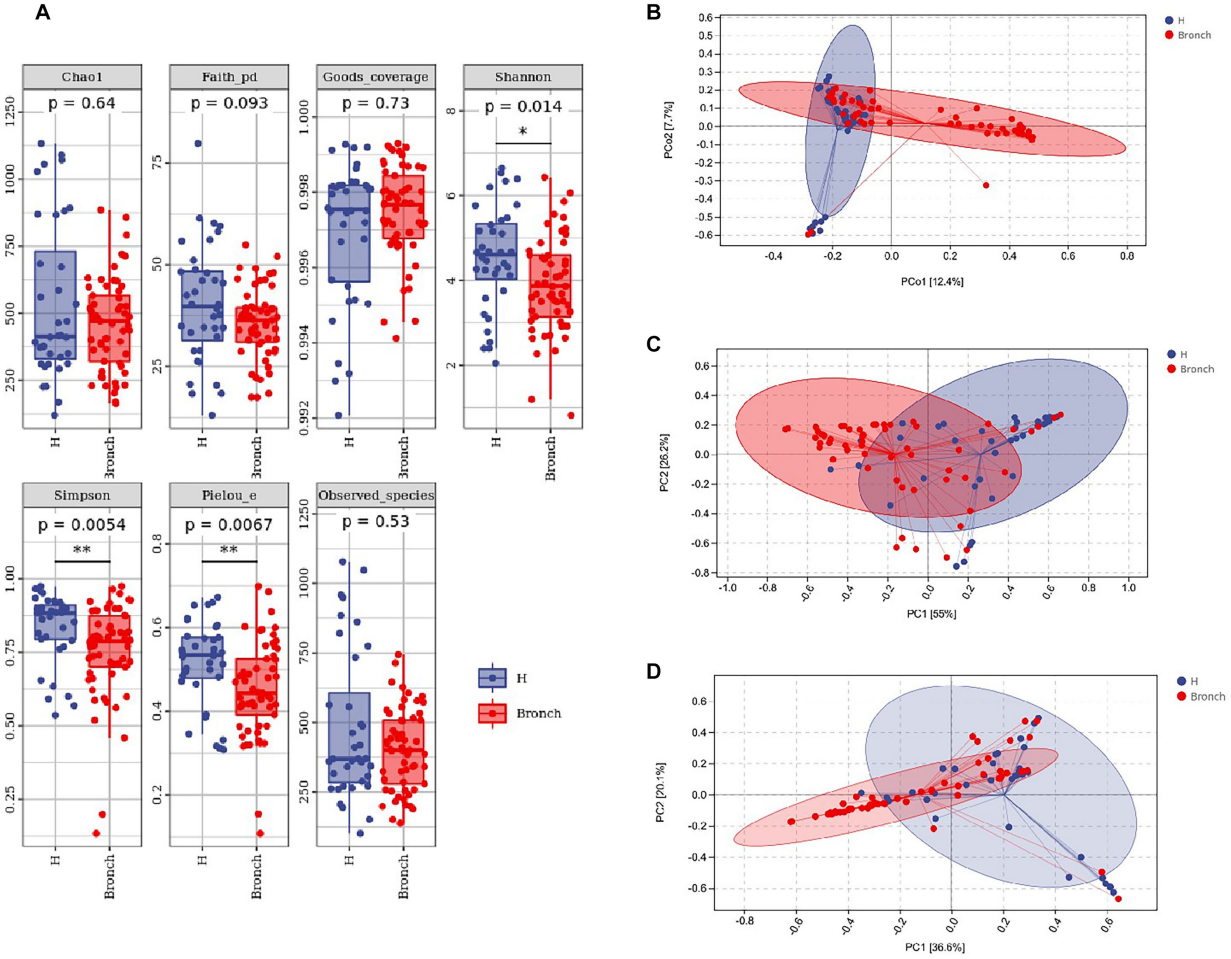
**(A)** Alpha diversity index analysis of the two groups of gut microbiota. **(B)** Principal coordinates analysis (PCoA) showing overall colonization differences between the two groups of samples. **(C)** Phylum-level colonization differences between the two groups of samples. **(D)** Genus-level colonization differences between the two groups of samples. *p < 0.05, **p < 0.01.

We analyzed the differences in the alpha diversity of intestinal microbiota between the two groups of children by the Mann–Whitney U test, and the results showed that the alpha diversity Shannon, Simpson and Pielou’s evenness indices at the OTU level were significantly different in the healthy group compared to the group with bronchiolitis. The results suggested that the diversity of intestinal microbiota in children with acute bronchiolitis was lower than those in healthy children (Figure 1A). We analyzed the differences in the overall microbial composition of the microbiota of the two groups using principal coordinate analysis (PCoA) and the differences in colonization at the phylum and genus levels between the two groups of samples using PCA. The results showed that there were differences in the overall species composition of the intestinal microbiota of children with acute bronchiolitis and healthy children (PERMANOVA *p* = 0.001) (Figure 1B), and there were differences in the composition of colonization at both the phylum and genus levels between the two groups (PERMANOVA *p* = 0.001) (Figures 1C,D).

### 3.3. The dominant OTUs at the phylum and genus levels in both groups of samples

**Figure 2:**
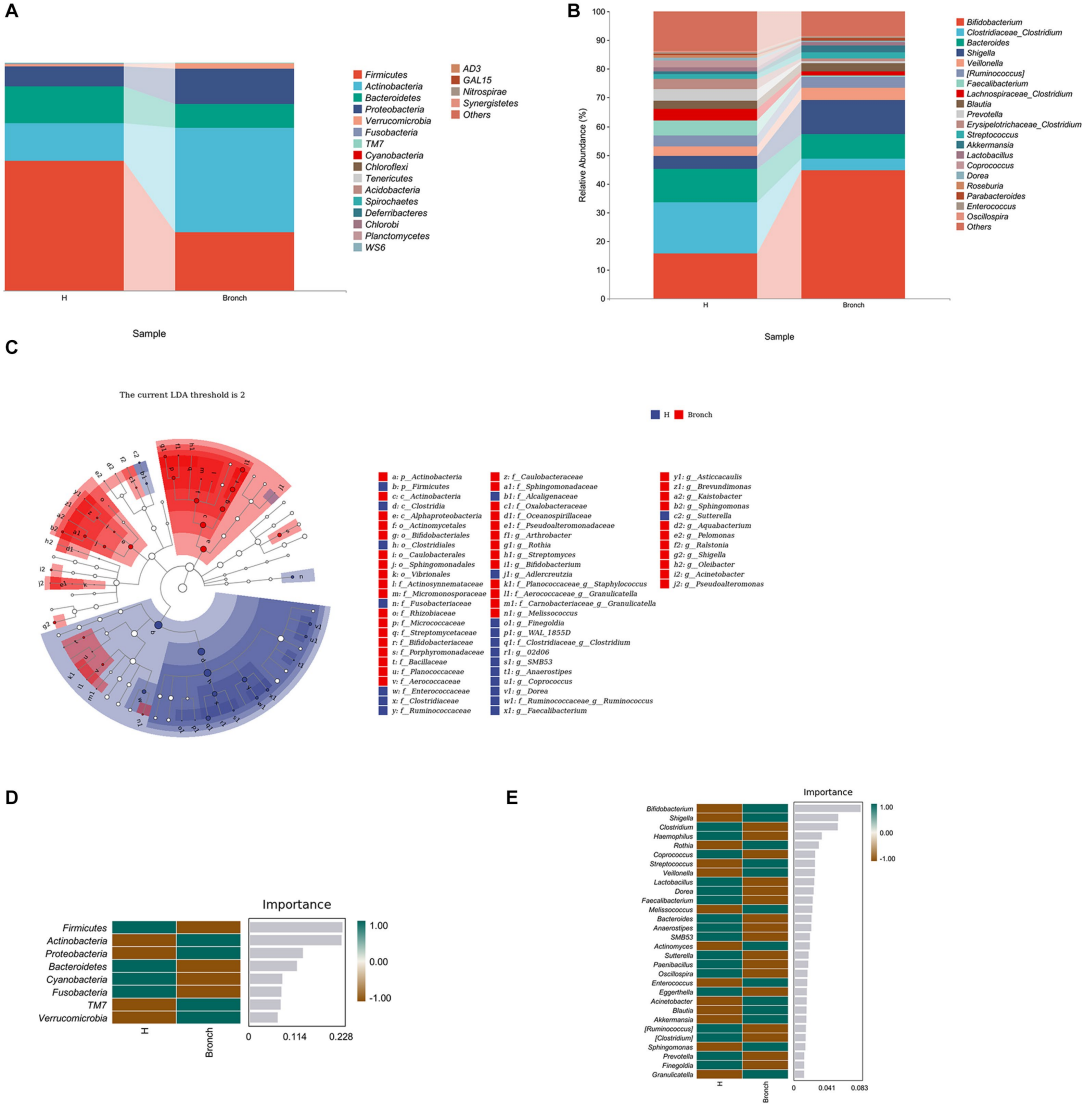
**(A)** Variation and differences in microbial composition at the phylum level. **(B)** Variation and differences in microbial composition at the genus level. **(C)** LEfSe enrichment analysis showing stable difference marker OTUs between the two groups. **(D)** Random forest analysis showing key marker OTUs between the two groups at the phylum level. **(E)** Random forest analysis showing key marker OTUs between the two groups at the genus level.

Multivariate cluster and LEfSe enrichment analyses based on the level of bacterial OTUs were used to identify stable difference marker OTUs between the bronchiolitis group and the healthy children group, and then their significance was determined by the Wilcoxon rank-sum test. Fifty-two OTUs were found to be significantly enriched in the bronchiolitis group, and 34 OTUs were significantly enriched in healthy children (LEfSe: *p* < 0.05, *q* < 0.1, LDA > 2.0) (Figures 2A–C). Subsequently, we further performed random forest analysis by calling the “classify_samples_ncv” function in the q2-sample classifier and nested stratified cross-tests to find the top 30 key marker OTUs in terms of abundance at the phylum and genus levels. The results showed that the intestinal microbiota of acute bronchiolitis had increased Actinobacteria, Proteobacteria, TM7, and Verrucomicrobia and decreased Firmicutes, Bacteroidetes, Cyanobacteria, and Fusobacteria at the phylum level compared to the intestinal microbiota of healthy children (Figure 2D). At the genus level, Bifidobacterium, Shigella, Rothia, Streptococcus, Veillonella, Melissococcus, Actinomyces, Enterococcus, Acinetobacter, Blautia Akkermansia, Sphingomonas, and Granulicatella were increased, and Clostridium, Haemophilus, Coprococcus, Lactobacillus, Dorea, Faecalibacterium, Bacteroides Anaerostipes, SMB53, Sutterella, Paenibacillus, Oscillospira, Eggerthella, [Ruminococcus], [Clostridium], Prevotella, and Finegoldia were decreased (Figure 2E).

### 3.4. Untargeted metabolite detection and KEGG enrichment analysis between the two groups

**Figure 3:**
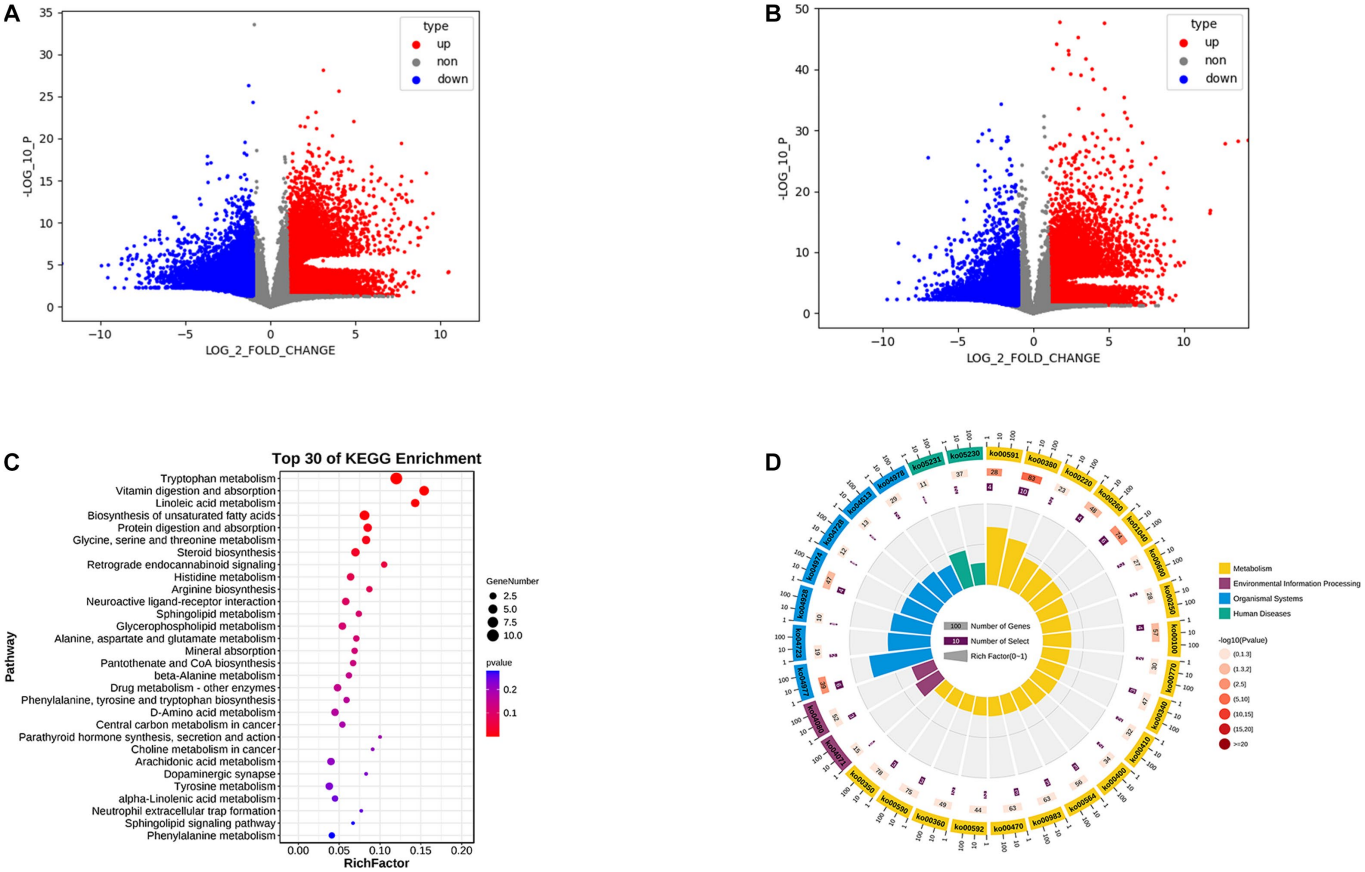
**(A)** Positive ion column differential metabolite volcano plot. **(B)** Negative ion column differential metabolite volcano plot. **(C)** KEGG enrichment analysis bubble plot. **(D)** KEGG enrichment analysis circle plot.

We performed OPLS-DA using pyopls, in which the total horizontal coordinate of the score plot represents the orthogonal first principal component and the vertical coordinate represents the first principal component. We used fold change analysis and a T test to analyze the two groups of samples for differential metabolites and found that 419 differential metabolites were obtained from negative ion column screening and 272 differential metabolites were obtained from positive ion column screening (FC > 2.0, *p* value <0.05, VIP > 1) (Figures 3A,B). Subsequently, we subjected the obtained metabolites to KEGG enrichment analysis, and a total of 67 differential signaling pathways were obtained. Among the first 30 pathways, 19 were associated with metabolism, 2 with human diseases, 2 with environmental information processing, and 7 with organismal systems (Figures 3C,D). We further analyzed their secondary pathways and found that 11 of the 19 metabolism-related pathways were amino acid metabolic pathways, 6 were lipid metabolic pathways, 1 was a xenobiotic biodegradation and metabolism pathway and 1 related to the metabolism of cofactors. Of the 7 pathways related to organismal systems, 2 were neurological, 3 were digestive, 1 was immune and 1 was endocrine.

### 3.5. KEGG network association analysis and intestinal microbiota and stool metabolites

**Figure 4:**
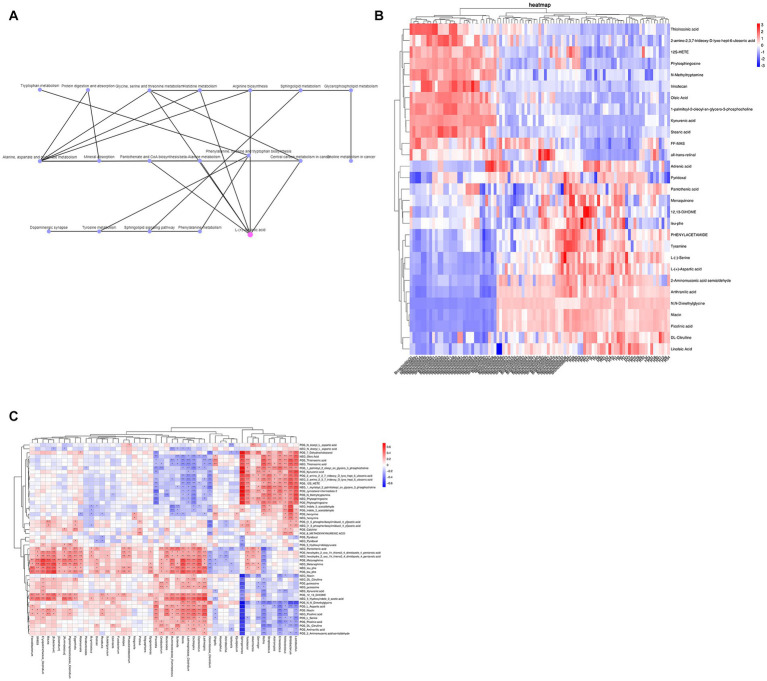
**(A)** KEGG network association analysis. **(B)** KEGG enrichment analysis of the top 30 pathway-associated metabolites. **(C)** Spearman rank correlation between metabolites and the abundance of the top 50 intestinal microbiota at the genus level.

By KEGG network association analysis, we further identified that the core pathways in the top 30 pathways may be alanine, aspartate and glutamate metabolism and glycine, serine and threonine metabolism pathways, while the key metabolite may be L-aspartic acid (which is enriched in several metabolic pathways, including arginine biosynthesis; alanine, aspartate and glutamate metabolism; glycine, serine and threonine metabolism; cysteine and methionine metabolism; histidine metabolism; beta-alanine metabolism; and D-amino acid metabolism) (Figure 4A). Subsequently, to combine the changes in gut microbiota and metabolic abnormalities, we screened a total of 48 metabolites (including 2 SCFAs butyric acid and acetic acid, phytosphingosine and L-aspartic acid) associated with the top 30 pathways of KEGG enrichment analysis (Figure 4B) and calculated the concentration of metabolites in feces in relation to the genus of intestinal microbiota. Spearman rank correlations were calculated between the concentrations of metabolites in feces and the top 50 species of genus abundance of intestinal microbiota, and association network analysis was used to find the key species (*R* > 0.6, *p* < 0.05). Forty-seven of the 48 metabolites were found to be correlated with the abundance of the top 50 species at the genus level, and the association network analysis identified Sphingomonas as the key species, which was correlated with 38 metabolites, including zymosterol-intermediate2, N-methyltryptamine, 1-palmitoyl-2-oleoyl-sn-glycero-3-phosphocholine, 1-myristoyl-2-palmitoleoyl-sn-glycero-3-phosphocholine, 7-dehydrocholesterol, stearic acid, kynurenic acid, and phytosphingosine, which were significantly positively correlated with L-serine, N–N-dimethylglycine, linoleic acid, L-aspartic acid and niacin (Figure 4C).

### 3.6. Correlation analysis of serum inflammatory factor assay levels

**Figure 5:**
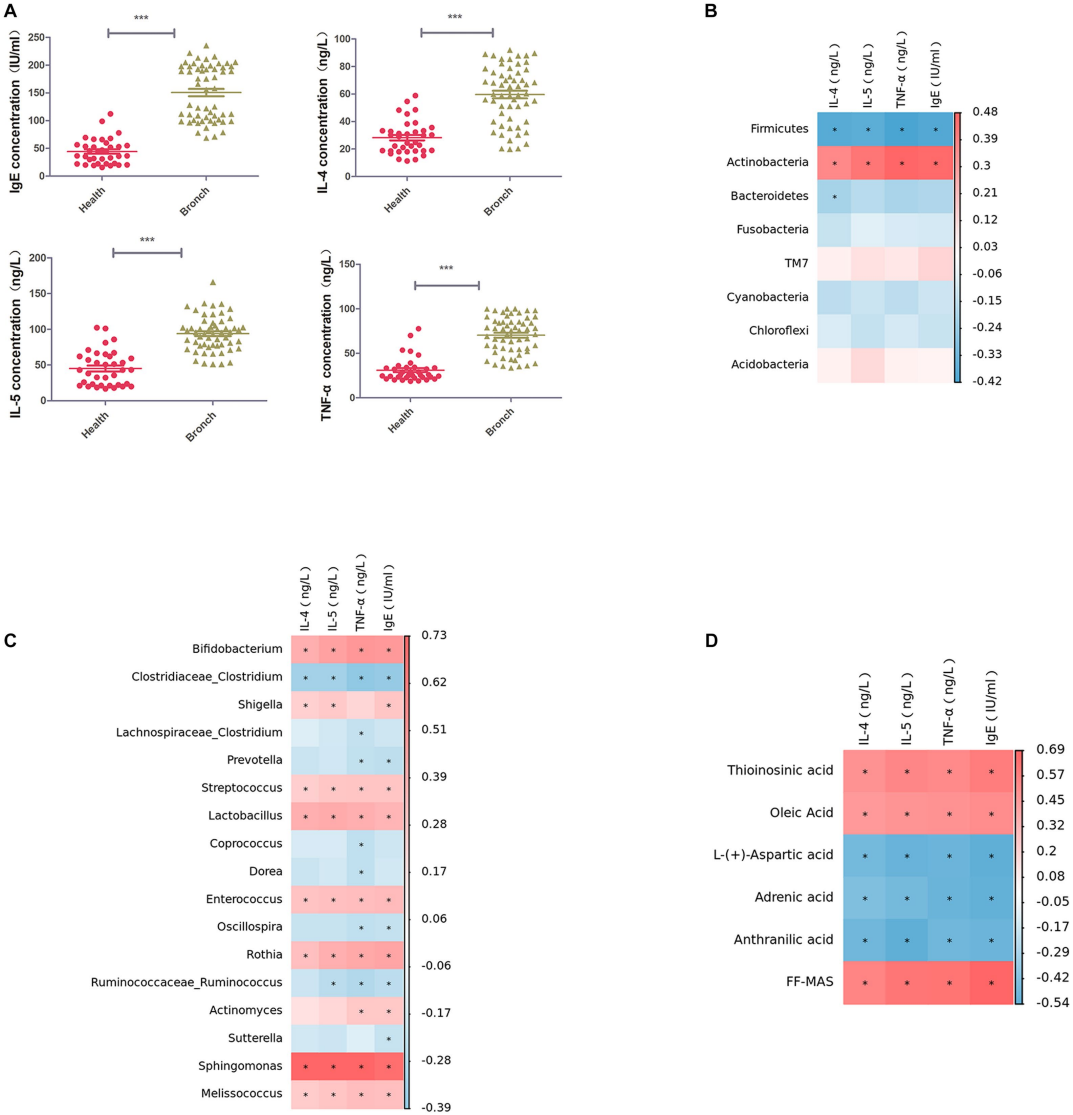
**(A)** ELISA for IL-4, IL-5, TNF-α, and IgE in serum. **(B)** Spearman rank correlation between IL-4, IL-5, TNF-α, and IgE in serum and the abundance of the top 50 strains of intestinal microbiota at the phylum level. **(C)** Spearman rank correlation between IL-4, IL-5, TNF-α, and IgE in serum and the abundance of the top 50 strains of intestinal microbiota at the phylum level. Spearman’s rank correlation between strains. **(D)** Spearman’s rank correlation between serum IL-4, IL-5, TNF-α, IgE, and intestinal metabolites. ***p < 0.001.

We used ELISA to detect IL-4, IL-5, TNF-α, and IgE in the serum of children in both groups, and the results suggested that IL-4, IL-5, TNF-α, and IgE were significantly elevated in children with bronchiolitis compared with healthy children (Figure 5A). Further Spearman correlation analysis (*R* > 0.6, *p* < 0.05) showed that IL-4, IL-5, TNF-α, and IgE were significantly negatively correlated with the intestinal microbiota phylum Firmicutes and significantly positively correlated with Actinobacteria (Figure 5B). IL-4, IL-5, TNF-α, and IgE were significantly negatively correlated with the intestinal microbiota genus levels. Clostridium was significantly negatively correlated with Bifidobacterium, Rothia, Streptococcus, Enterococcus, Sphingomonas, Granulicatella, and Lactobacillus, with Sphingomonas having the highest correlation (Figure 5C). IL-4, IL-5, TNF-α, and IgE were negatively correlated with the intestinal metabolites picolinic acid and L-aspartic acid and positively correlated with FF-MAS and thioinosinic acid (Figure 5D).

### 3.7. Metabolite ROC analysis

**Figure 6:**
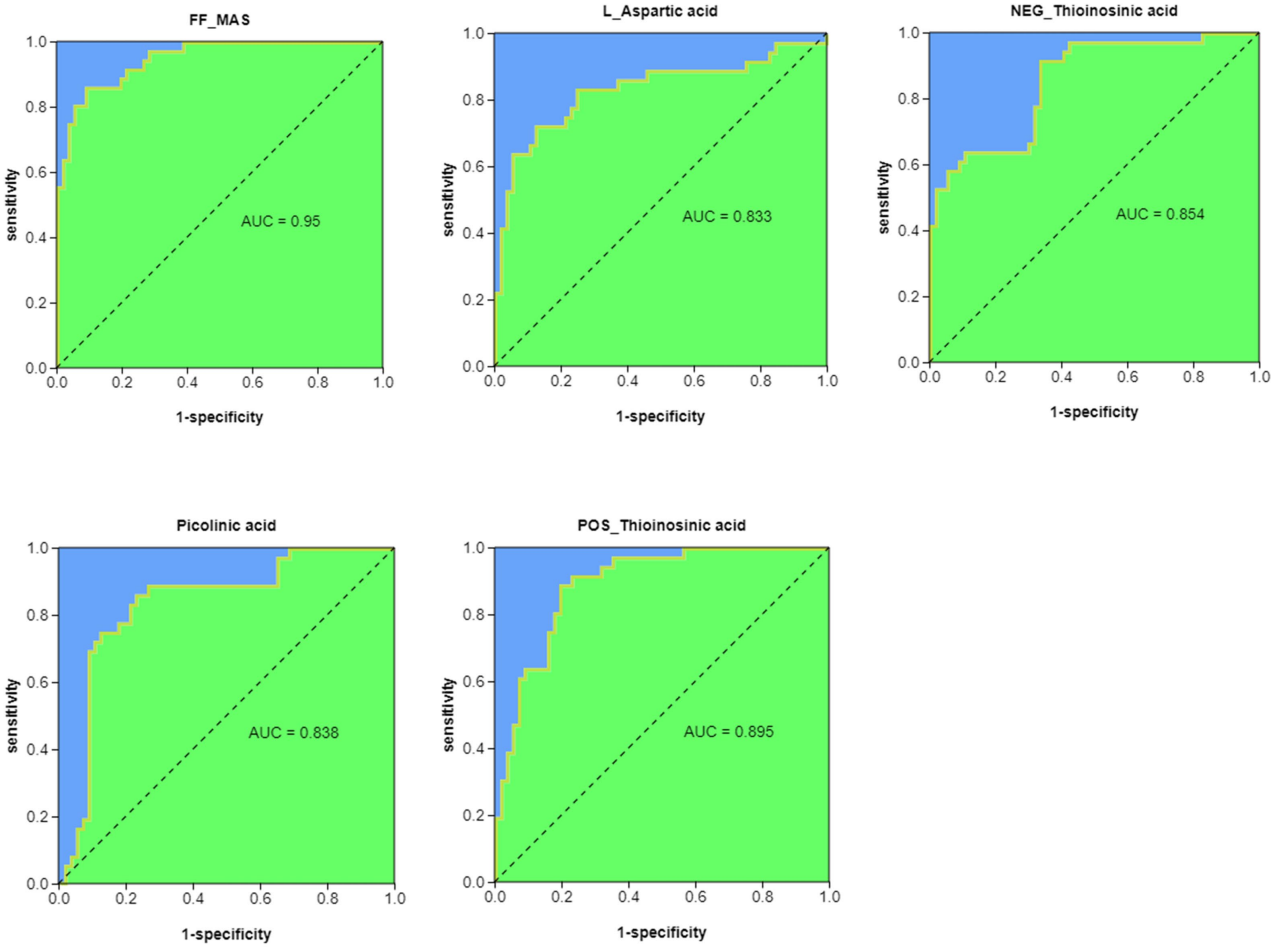
ROC analysis of potential biomarkers for differentiating healthy children from children with acute bronchiolitis.

We performed ROC analysis (AUC > 0.8) on the top 30 pathway-related metabolites of KEGG enrichment analysis and found that FF-MAS, L-aspartic acid, thioinosinic acid, and picolinic acid were all well distinguished between healthy children and children with acute bronchiolitis (Figure 6).

### 3.8. Establishment of an RSV-infected mouse model and the therapeutic effect of Clostridium

**Figure 7:**
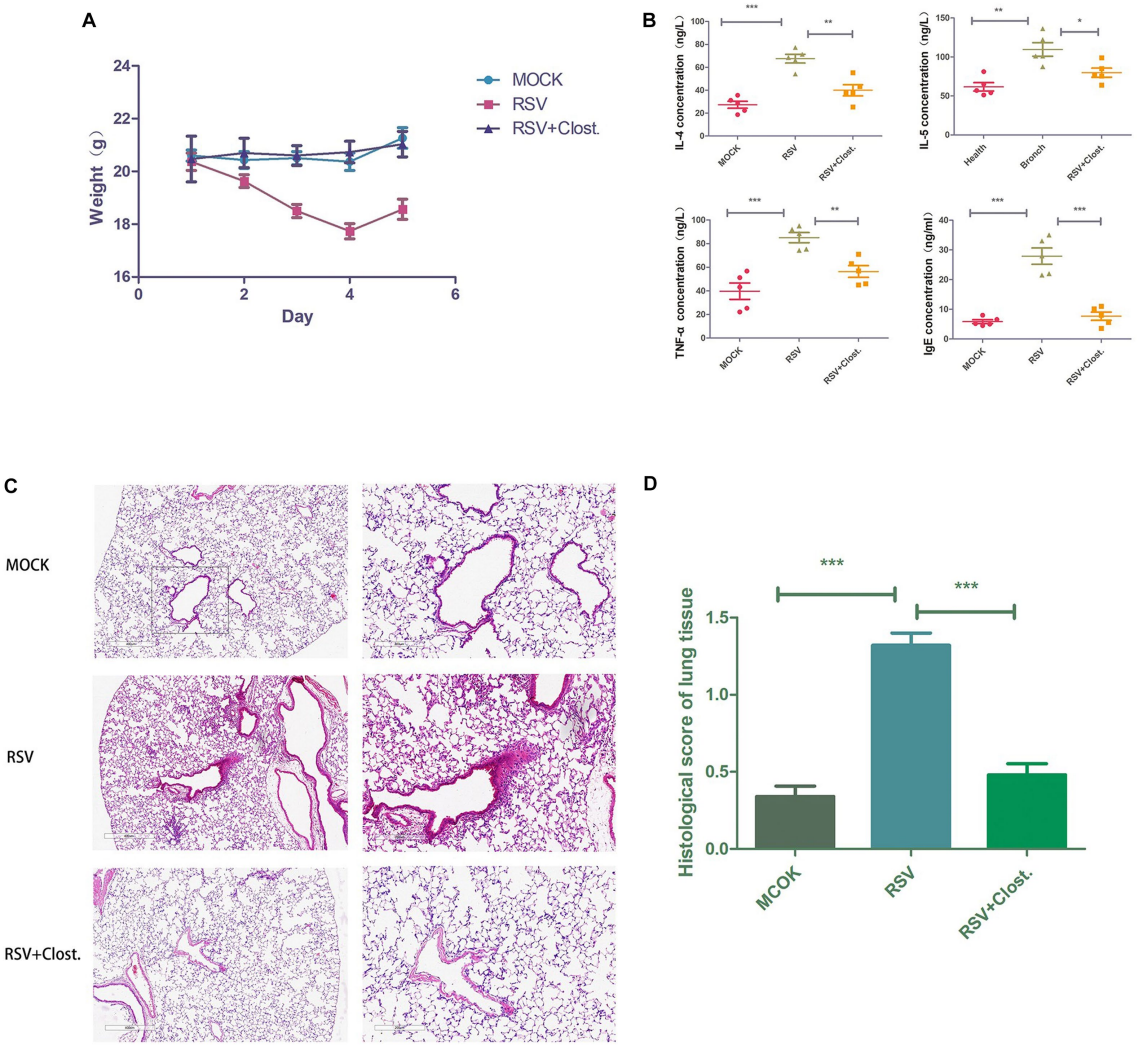
**(A)** Body weight change curves of the three groups of mice. **(B)** Results of IL-4, IL-5, TNF-α, and IgE assays in the serum of the three groups of mice. **(C)** Histopathological sections of lungs of the three groups of mice. **(D)** Inflammation scores of lung tissues of the three groups of mice. *p < 0.05, **p < 0.01, ***p < 0.001.

To further support our findings, we established an RSV-infected mouse model using RSV with the addition of *Clostridium butyricum* for adjuvant treatment and found that RSV-infected mice with the addition of *Clostridium butyricum* showed significant improvements in body weight, lung histopathological changes, and serum inflammatory factors compared to the control group (Figure 7).

## 4. Discussion

Our clinical case study of 36 healthy children and 57 children with bronchiolitis revealed that obesity, passive smoking, a family history of allergic disease, a history of eczema, and conception by *in vitro* fertilization were high risk factors for the development of bronchiolitis, while place of residence and breastfeeding were protective factors (Table 1). Obesity leads to adipocyte hypertrophy and increased macrophages, and the increase in macrophages facilitates the production of gene products of the proinflammatory environment; adipocytes can secrete the proinflammatory cytokines IL-6, hs-CRP, and TNF-α, leading to an inflammatory state in the organism (Cox et al., 2015). In addition, previous studies have also reported an association between obesity and the development of bronchiolitis (Teijeiro and Gómez, 2021). The process of passive smoking in children can lead to damage to airway cilia and increased airway resistance, which can increase the risk of bronchiolitis (Pavić et al., 2014). Several studies have shown that the occurrence of allergic diseases is significantly associated with genetic factors, with a 50% prevalence of allergic diseases in children whose parents are both allergic (Thomsen et al., 2012). The development of bronchiolitis is associated with allergic diseases, and children with a history of eczema have increased levels of IgE, which is likely to be a major trigger for further development of asthma. According to the hygiene hypothesis, children living in rural areas, whose early exposure to various microorganisms is high, are more favorable for the establishment of immune homeostasis and have a reduced risk of developing allergic diseases. Breast milk contains high levels of lysozyme and immune factors that significantly reduce the production of IgE in the organism, which in turn reduces the chances of bronchiolitis (Iyengar and Walker, 2012). In a retrospective study of medical records of more than 100 infants under one year of age hospitalized with RSV infection, the authors found that breastfed infants had milder symptoms and a reduced need for oxygen therapy than formula-fed infants (Jang et al., 2020). Another similar study also found that hospitalized breastfed infants with RSV infection had shorter hospital stays (Nishimura et al., 2009). These studies show a strong relationship between alterations in the gut microbiome early in life and lung disease. In addition, with the introduction of IVF-ET technology in recent years, an increasing number of IVF babies are being born, but the incidence of multiple births in IVF babies is high (Fiddelers et al., 2007). The establishment of intestinal microbiota in the early stage of IVF is not good, which may be one of the reasons why IVF infants are prone to allergic diseases such as bronchiolitis. This needs to be verified by a large number of samples in the future.

We performed a differential analysis of the alpha diversity of the intestinal microbiota in children and found that the intestinal microbiota diversity was significantly reduced in children with bronchiolitis compared with the intestinal microbiota of healthy children, and there were significant differences in the overall colonization (Figure 1). The results are consistent with the findings of Hasegawa et al. (2016) that the microbiota richness between groups represents the complexity of the population’s bacterial ecology. In general, the higher the abundance of microbiota is, the better the ecology of the population and the stronger the ability to resist invasion by foreign bacteria or other microorganisms; however, the causal relationship is still unclear. With reference to our previous clinical data analysis, factors that influence the development of acute bronchiolitis, such as obesity, passive smoking, family history of allergy, history of eczema, IVF and breastfeeding, may also affect the intestinal microbiota of children; therefore, we speculate that changes in the intestinal microbiota of children with acute bronchiolitis may have occurred before the onset of the disease. In other words, the alteration of intestinal microbiota may be one of the important triggers for the development of acute bronchiolitis, which needs to be verified in future studies. The diversity of intestinal microbiota is important for the health and growth of children, including the development of immune imbalance, malnutrition, autism, inflammatory bowel disease, diabetes, and allergies, all of which have been found to be associated with intestinal microbiota imbalance, and Korten et al. (2016) showed that micromicrobiota alterations were still present 3 weeks after viral infection, so we speculate that the increased incidence of future asthma in children with acute bronchiolitis may also be related to alterations in the gut microbiota.

We further searched for stable differential species by multivariate cluster analysis, LEfSe enrichment analysis, and random forest analysis and found that children with acute bronchiolitis had increased gate levels of Actinobacteria, Proteobacteria, etc., and increased Firmicutes, Bacteroidetes, etc., compared to healthy children. Cyanobacteria, Fusobacteria, etc., were decreased (2A, 2C, 2D). At the genus level, Clostridium and others were decreased, and Bifidobacterium, Sphingomonas, Shigella, Rothia, Streptococcus, Veillonella and others were increased (Figures 2B,C,E). The bacteria in the intestine mainly include Bacteroidetes, Firmicutes, Proteobacteria, and Actinobacteria, with Bacteroidetes predominating in adults and Firmicutes predominating in normal infants and children. The populations of both Firmicutes and Bacteroidetes increase with age, and both are the main species producing SCFAs. In recent years, the regulatory role of SCFAs in inflammation has received increasing attention. SCFAs can maintain intestinal homeostasis and suppress inflammation by inducing the differentiation of intestinal naïve CD4+ T cells to regulatory T cells (Luu and Visekruna, 2019). Possible mechanisms include inhibition of histone deacetylase activity, enhancement of acetylation levels of forkhead box p3, a key transcription factor of Treg, and secretion of IL-10 by dendritic cells and macrophages through GPR109, a receptor on dendritic cells and macrophages (Bajic et al., 2020). Transforming growth factor-β induces the differentiation of initial CD4+ T cells to Tregs and thus regulates inflammation. Therefore, we speculate that the altered intestinal microbiota in acute bronchiolitis likely further influences the secretion of SCFAs, which may affect subsequent disease regression and long-term prognosis. Studies have demonstrated that both RSV and influenza infection in mice impact the gut microbiota composition (Groves et al., 2018), rendering these mice more susceptible to subsequent enteric infection (Yildiz et al., 2018). Influenza-induced gut microbial changes and reduced production of SCFAs were also shown to increase susceptibility to secondary pneumococcal infection (Sencio et al., 2020), underscoring the intense crosstalk between the gut and lung.

At the genus level, Clostridium belongs to the phylum Firmicutes, and the results of LEfSe enrichment analysis showed that the decrease in the phylum Firmicutes was mainly due to a decrease in Clostridium at the genus level. Further Spearman correlation analysis found that IL-4, IL-5, TNF-α, and IgE were significantly negatively correlated with Clostridium and significantly positively correlated with Sphingomonas at the genus level of intestinal microbiota (Figure 5C). Moreover, metabolite and intestinal microbiota association network analysis identified Sphingomonas as the key species, which was correlated with 38 metabolites. This suggests that Clostridium and Sphingomonas may be key strains influencing the disease progression of bronchiolitis, and the discovery of this key strain has the potential to provide new microbiota markers and targets for the future treatment of bronchiolitis. Clostridium is a popular strain currently under study, and it has been shown to significantly inhibit food allergy-induced intestinal Clostridium, which has been shown to significantly inhibit the inflammatory response to food allergies and contribute to the repair of the intestinal mucosal barrier (Huang et al., 2016). Clostridium is a very important butyric acid-producing bacterium. Butyric acid is a type of short-chain fatty acid, and some studies have found that the levels of butyric acid are significantly lower in the intestinal microbiota of allergic children. Butyric acid can contribute to the activation of the immune system, accelerate the removal of proinflammatory factors and repair damaged tissue cells. Its anti-inflammatory mechanism is mainly through affecting the release of immunomodulatory inflammatory cytokines (IL-2, IL-4, IL-8, TNF-α). The anti-inflammatory mechanism exerts its immune function by affecting the release of immunomodulatory inflammatory cytokines (IL-2, IL-4, IL-8, TNF-α) (Markle et al., 2013). Zhu et al. (2021) found that Clostridium could modulate macrophage polarization and the secretion of various inflammatory factors through the metabolite butyrate, thereby reducing lung inflammation caused by viral infection. In combination with our finding of abnormal butyrate metabolism in children with bronchiolitis by KEGG enrichment analysis (Figure 4B), we consider that a decrease in Clostridium leads to a decrease in organismal butyrate production, which exacerbates pulmonary inflammation caused by viral infection by increasing macrophage polarization and inflammatory factor production. Sphingomonas is a gram-negative bacterium that can reduce lung inflammation by activating the sphingolipid Sphingomonas, which is a gram-negative bacterium that increases sphingolipid biosynthesis by activating serine palmitoyltransferase (SPT, EC), a key enzyme for sphingolipid synthesis and metabolism (Li et al., 2020). Sphingolipids have been a hot topic of research in the pathogenesis of bronchiolitis in recent years, and their metabolite sphingosine 1-phosphate (S1P) may play an important role in the pathophysiological alterations of lung disease by participating in a variety of cellular inflammatory mechanisms through the S1P/SPHK (sphingosine kinase) signaling pathway, which regulates mast cell activation and the release of inflammatory factors (Tran et al., 2016; Li et al., 2020). Stewart et al. (2017) and Hasegawa et al. (2017) have shown that abnormalities in sphingolipid metabolism may be importantly associated with the development of capillary bronchiectasis. Unfortunately, we were not able to perform further assays of liposomes, which is what we plan to do next. Another interesting finding of our study is that the increase in bifidobacteria was positively associated with acute bronchiolitis inflammatory factors (Figure 5C). In recent years, studies on bifidobacteria and allergic and infectious diseases in children have increased, with most of the results suggesting a positive effect of bifidobacteria on disease and a few studies finding no beneficial effect of bifidobacteria supplementation on disease recovery. We speculate that the balance of human intestinal microecology is very important and that any disturbance of bacterial species (including over supplementation of probiotics) may have health consequences. Our current understanding of intestinal microecology is still inadequate, and blind probiotic supplementation for diseases may be counterproductive, which requires us to conduct intestinal microbiota testing for different diseases to find key strains and target probiotic supplementation therapy.

Meanwhile, the KEGG enrichment analysis further verified our previous speculation that 11 of the 19 differential metabolic pathways were amino acid metabolic pathways and 6 were lipid metabolic pathways, suggesting that amino acid and lipid metabolism may have important roles in the progression of acute bronchiolitis (Figures 3E,F). Interestingly, we also found enrichments in two neurological-related pathways; does this imply that the onset of acute bronchiolitis has an impact on neurological development in children? This possibility needs to be further investigated and confirmed in follow-up studies.

We previously mentioned butyrate among the 48 differential metabolites and linked it to a decrease in Clostridium, while another SCFA, acetic acid, also showed a decrease in children with acute bronchiolitis (Figure 4B). Acetic acid is an inhibitor of histone deacetylases (HDACs), and Kendrick et al. (2010) found in acetic acid-treated human macrophages that acetate induced high histone acetylation by inhibiting HDAC activity and was associated with reduced secretion of inflammatory factors such as IL-6, IL-8, and TNF-α in macrophages. In addition, it was found that acetic acid is associated with the development of asthma; acetic acid can regulate the onset and development of asthma by activating the GPR43 receptor to inhibit HDAC9 activity, increasing the transcriptional level of the Foxp3 gene, and promoting Treg cell proliferation and immunosuppressive function (Zeng and Chi, 2015). Therefore, we considered that the decrease in acetic acid may also be associated with the progression of acute bronchiolitis. Thus, butyric acid, acetic acid and sphingolipids are likely new targets for the treatment of acute bronchiolitis. In addition, differential metabolite ROC analysis revealed that FF-MAS, L-aspartic acid, thioinosinic acid, and picolinic acid all distinguished healthy children from children with acute bronchiolitis (Figure 6); these metabolic biomarkers are not currently reported in acute bronchiolitis and asthma. In the future, these metabolic biomarkers may be used as new biomarkers for the diagnosis of acute bronchiolitis.

To further validate our previous speculations and clarify the critical role of Clostridium reduction in acute bronchiolitis, we performed *Clostridium butyricum* supplementation in RSV-infected model mice. We found that supplementation with *Clostridium butyricum* significantly reduced RSV-induced lung infection and decreased the secretion of the inflammatory factors IL-4, IL-5, TNF-α, and IgE. In children with asthma, *Clostridium butyricum* alleviated airway inflammation and pulmonary resistance by inhibiting specific IgE expression and mast cell degranulation (Juan et al., 2017). In addition, *Clostridium butyricum* has been reported to regulate the production of inflammatory factors in macrophages by inhibiting the NF-κB and ERK signaling pathways through the metabolite butyric acid (Park et al., 2019). As TNF-α is an upstream factor of the NF-κB pathway, we speculate that *Clostridium butyricum* may inhibit the activation of the NF-κB pathway through the downregulation of TNF-α by the metabolite butyric acid, which in turn inhibits RSV infection-induced lung inflammation.

In conclusion, our study identified significant differences in intestinal microbiota and metabolites between children with acute bronchiolitis and healthy children, identified key strains of bacteria and signature metabolites, and validated the therapeutic effect of probiotic addition in acute bronchiolitis. However, there are some limitations of our study, on the one hand, the small sample size leads to low credibility of the study, and on the other hand, infant age needs to be considered for the analysis because the period between 3 months and 2 years shows a very dynamic stool microbiome development. As these features are measured during acute bronchiolitis, we cannot conclude on cause or consequence. The gut microbial composition is complex, and the exploration of the interactions between the microbiota and the host is still in its infancy. These interactions are extremely complex, and any local changes in them may have an impact on the health of the organism. At this stage, only three aspects of the gut microbiome are known to be involved in the process of airway allergic diseases and to influence their development: microecology, genus, and metabolites of microorganisms. The specific mechanisms of action between them are still not fully elucidated. In addition, the new concept of the “enteropulmonary axis” is at a completely preliminary stage, and there are no other studies or reports on the relative importance of the intestinal and pulmonary microbiota in assisting the body to resist disease. Nevertheless, they are still a hot topic of research now and in the future, and a number of experts and scholars are already working in this area, aiming to reveal the complex mechanisms between them. With the continuous updating of sample processing methods, advancement of sequencing technologies and increasing depth of interpretation of sequencing results, significant breakthroughs will be made in this field in the future. Moreover, using intestinal microbiota as a new target will provide a safer and more effective method for the prevention and treatment of airway allergic diseases and will provide new ideas for the development of more novel drugs, which is of great significance for the future development of medicine.

## Data availability statement

The datasets presented in this study can be found in online repositories. The names of the repository/repositories and accession number(s) can be found below: https://www.ncbi.nlm.nih.gov/, PRJNA898847.

## Ethics statement

The studies involving human participants and animal study were reviewed and approved by Chongqing Health Center for Women and Children Ethics Committee. Written informed consent to participate in this study was provided by the participants’ legal guardian/next of kin and was obtained from the individual(s), and minor(s)’ legal guardian/next of kin, for the publication of any potentially identifiable images or data included in this article.

## Author contributions

X-bW, JW, and YT wrote the manuscript. JJ and X-mL reviewed the manuscript. All authors have read and approved the manuscript.

## Funding

This study was supported by the Chongqing medical scientific research project (Joint project of Chongqing Health Commission and Science and Technology Bureau) (no. 2019ZDXM017), Natural Science Foundation of Chongqing (no. cstc2020jcyj-msxmX0327), and Chongqing Yuzhong District’s Fundamental Research and Frontiers Exploration Project (no. 20200146).

## Conflict of interest

The authors declare that the research was conducted in the absence of any commercial or financial relationships that could be construed as a potential conflict of interest.

## Publisher’s note

All claims expressed in this article are solely those of the authors and do not necessarily represent those of their affiliated organizations, or those of the publisher, the editors and the reviewers. Any product that may be evaluated in this article, or claim that may be made by its manufacturer, is not guaranteed or endorsed by the publisher.

## Footnotes

^1^http://huttenhower.sph.harvard.edu/galaxy/

^2^https://www.genome.jp/kegg/

^3^https://www.metaboanalyst.ca/

## Abbreviations

FDR: false discovery rate
LefSe: linear discriminant analysis effect size
PCA: principal component analysis
OPLS-DA: latent structure orthogonal projection-discriminant analysis
RSV: respiratory Syncytial Virus
IVF-ET: *in vitro* fertilization and embryo transfer
SCFAs: Short chain fatty acids
SPT, EC: serine palmitoyltransferase
S1P: sphingosine-1-phosphate
SPHK: Sphingosine kinase
HDACs: histone deacetylases
TNF-α: tumor necrosis factor-α
